# Papillary Muscle Maneuvers: Pathophysiology-based Approach in Secondary Mitral Regurgitation

**DOI:** 10.31083/j.rcm2508283

**Published:** 2024-08-09

**Authors:** Evaldas Girdauskas, Sina Stock, Elisa Favot, Blerim Luani, Tatiana Sequeira-Gross, Christian Dumps, Maria von Stumm, Tamer Owais, Wolfgang von Scheidt

**Affiliations:** ^1^Department of Cardiothoracic Surgery, University Hospital Augsburg, 86150 Augsburg, Germany; ^2^Department of Cardiology, Hospital Ingolstadt, 85049 Ingolstadt, Germany; ^3^Department of Anesthesiology and Intensive Care, University Hospital Augsburg, 86150 Augsburg, Germany; ^4^Department of Congenital and Pediatric Cardiac Surgery, German Heart Center Munich, Technische Universität München, 80636 Munich, Germany; ^5^Division of Congenital and Pediatric Heart Surgery, University Hospital Munich, Ludwids-Maximilians-University, 80539 Munich, Germany; ^6^1. Medizinische Klinik, University Hospital Augsburg, 86150 Augsburg, Germany

**Keywords:** secondary mitral regurgitation, mitral valve repair, papillary muscle maneuvers

## Abstract

The treatment of secondary mitral regurgitation (SMR) remains challenging despite the implementation of modern heart failure medication and established catheter-based techniques. Only a subgroup of SMR patients benefit from mitral valve (MV) intervention, and the long-term prognostic benefit of different therapeutic approaches in SMR remains controversial. A literature search was conducted through PubMed and Embase databases to identify relevant studies addressing the pathophysiological background for papillary muscle maneuvers in SMR and currently available surgical techniques. Furthermore, the studies evaluating patients’ selection criteria for papillary muscle maneuvers were specifically considered. Articles were selected based on quality and relevance. Over the last two decades, papillary muscle maneuvers have evolved as a pathophysiology-based treatment strategy to address left ventricular (LV) remodeling in SMR. In particular, patients with severe leaflet tenting and moderate heart failure phenotype seem to benefit most from papillary muscle maneuvers that improve LV geometry and thereby the durability of MV repair. We conclude that papillary muscle maneuvers are an evolving pathophysiology-based treatment strategy of ventricular SMR which target papillary muscle displacement due to LV remodeling.

## 1. Introduction

Population-based data on secondary mitral regurgitation (SMR) are currently sparse, and published literature gives only a rough estimate of SMR prevalence. In a large cohort of patients with heart failure and reduced ejection fraction (HFrEF), mild-to-moderate SMR and severe SMR were found in 49% and 24%, respectively [1]. Following myocardial infarction, the prevalence of moderate-to-severe SMR was 12% and was associated with a 3-fold increase in heart failure and a 1.5-fold increase in death during a mean of 5-year follow-up period [2].

In addition to the negative prognostic impact of SMR in heart failure [1, 2, 3], SMR leads to a tremendous socioeconomic burden and resource utilization, which is due to repeated hospitalizations, home healthcare and costly medications [4]. Medically treated patients with SMR and heart failure require an average of 3.3 hospitalizations per year, resulting in an in-hospital stay of one month per year and a 1-year mortality of 15% [4]. Overall, mean annual costs of acute hospitalization and rehabilitation stays were €13.538 ± 11.692. This illustrates the medical and socioeconomic impact of SMR on the national healthcare system and highlights the clinical relevance of this underestimated and undertreated disease [5].

## 2. Methods

Articles discussed in this review were identified through a literature search of English language articles in PubMed (1946 to the present) and Embase (1974 to the present), last updated December 5, 2023. The search was limited to adult human studies and original articles, published in the English language. We focused on the identification of articles studying surgical treatment strategies of SMR and their outcomes. The initial literature screening was conducted using the following Medical Subject Headings (MeSH)-based terms: “mitral valve”, “mitral valve insufficiency” and “papillary muscles” in various combinations. The selection criteria for inclusion in the review was the exact description of the surgical technique used for SMR treatment (in particular, papillary muscle maneuvers) and their short- and/or long-term outcome analysis. One author (EG) screened all titles and abstracts to identify publications for full-text review. In cases of repetitive reports from a single institution, we critically looked at patient cohort details and aimed to select a single article based on the largest number of patients included. Potential full-text articles were evaluated independently by three authors. We selected the articles by group consensus based on quality and relevance. The reference lists of all selected full-text articles were screened to identify additional relevant studies and updates were continued until December 2023. Additionally, current articles were identified through additional searches of in-press articles in relevant cardiothoracic surgical journals.

## 3. Results

### 3.1 Atrial vs. Ventricular SMR

**Fig. 1.**
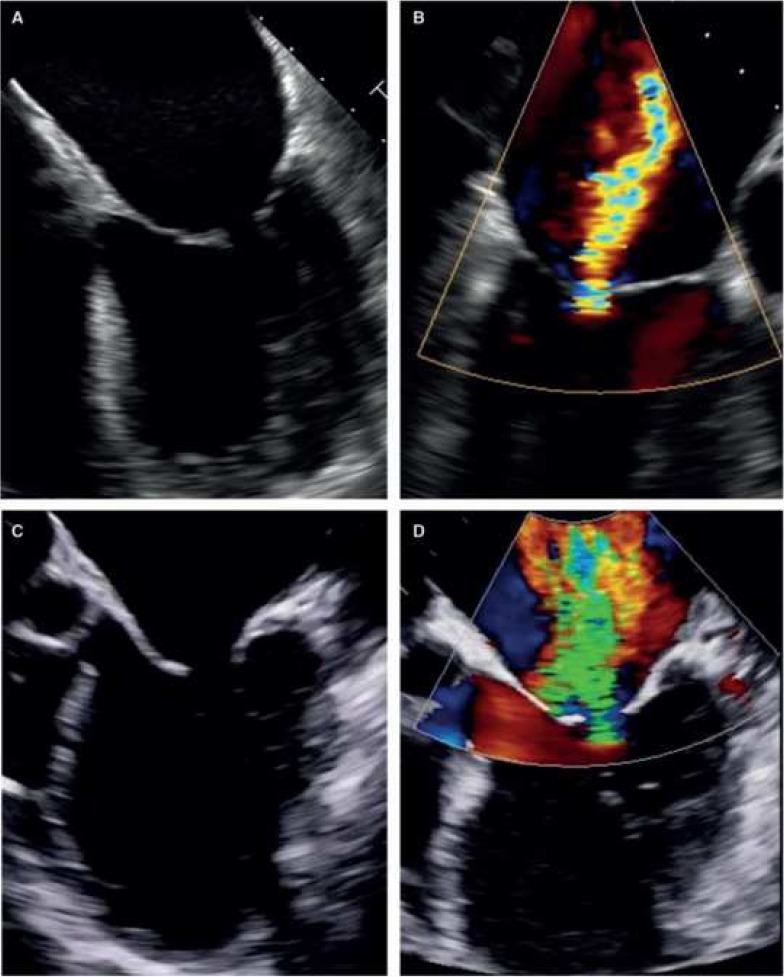
**Atrial and ventricular secondary mitral regurgitation (SMR)**. (A,B) atrial SMR with significant dilatation of mitral valve (MV) annulus, preserved left ventricular (LV) geometry and normal systolic motion of both MV leaflets. Central coaptation defect results in central regurgitation; (C,D) ventricular SMR caused by a significant LV remodeling, tenting of MV leaflets. Reduced systolic MV leaflet motion results in multiple complex, eccentric regurgitation jets (adapted from [10]).

SMR defines mitral regurgitation in the context of a structurally normal mitral valve to distinguish it from degenerative disease. SMR may occur due to predominant mitral valve (MV) annulus dilatation (i.e., atrial SMR) (Fig. 1A,B) or primary left ventricular (LV) disease, which leads to LV remodeling and systolic leaflet tenting (i.e., ventricular SMR) (Fig. 1C,D). Therefore, atrial and ventricular SMR indicate two different pathophysiological pathways in SMR development. Acknowledgment of this heterogeneity is crucial, since many previous publications considered SMR to be a single clinical entity. This heterogeneity of SMR and the individual mixture of atrial and ventricular SMR patients may explain, in part, the discrepancy in the outcomes of previous SMR trials.

### 3.2 Atrial SMR

Atrial SMR is common in the context of long-standing atrial fibrillation or heart failure with preserved ejection fraction (HFpEF) with concomitant left atrial dilatation [6]. The pathomechanism of atrial SMR is left atrial enlargement, leading to MV annulus dilatation with inadequate leaflet coaptation, while LV geometry and systolic LV function are typically preserved (i.e., so-called atrioventricular inversion).

Although the prevalence of atrial SMR is unknown, relevant mitral regurgitation has been reported in 4–7% of patients with persistent atrial fibrillation [7]. The critical element of atrial SMR treatment is MV annulus reduction by a surgical ring or catheter-based annuloplasty. Furthermore, the transcatheter edge to edge repair (TEER) procedure addressing the leaflet coaptation defect in MV annular dilatation has been successfully used in atrial SMR [8]. Due to the preserved LV geometry in atrial SMR, such patients show favorable clinical outcomes after surgical annuloplasty with a low mitral regurgitation (MR) recurrence compared to other types of SMR [9]. Therefore, we strongly recommend separating the patients with atrial SMR from the remaining SMR cohort to improve the comparability of treatment outcomes in SMR.

### 3.3 Ventricular SMR

Ventricular SMR results from an underlying LV disease leading to LV remodeling. Underlying LV diseases are manifold, including ischemic or non-ischemic cardiomyopathies, such as idiopathic dilated cardiomyopathy, valvular or toxic cardiomyopathy, or myocarditis.

**Fig. 2.**
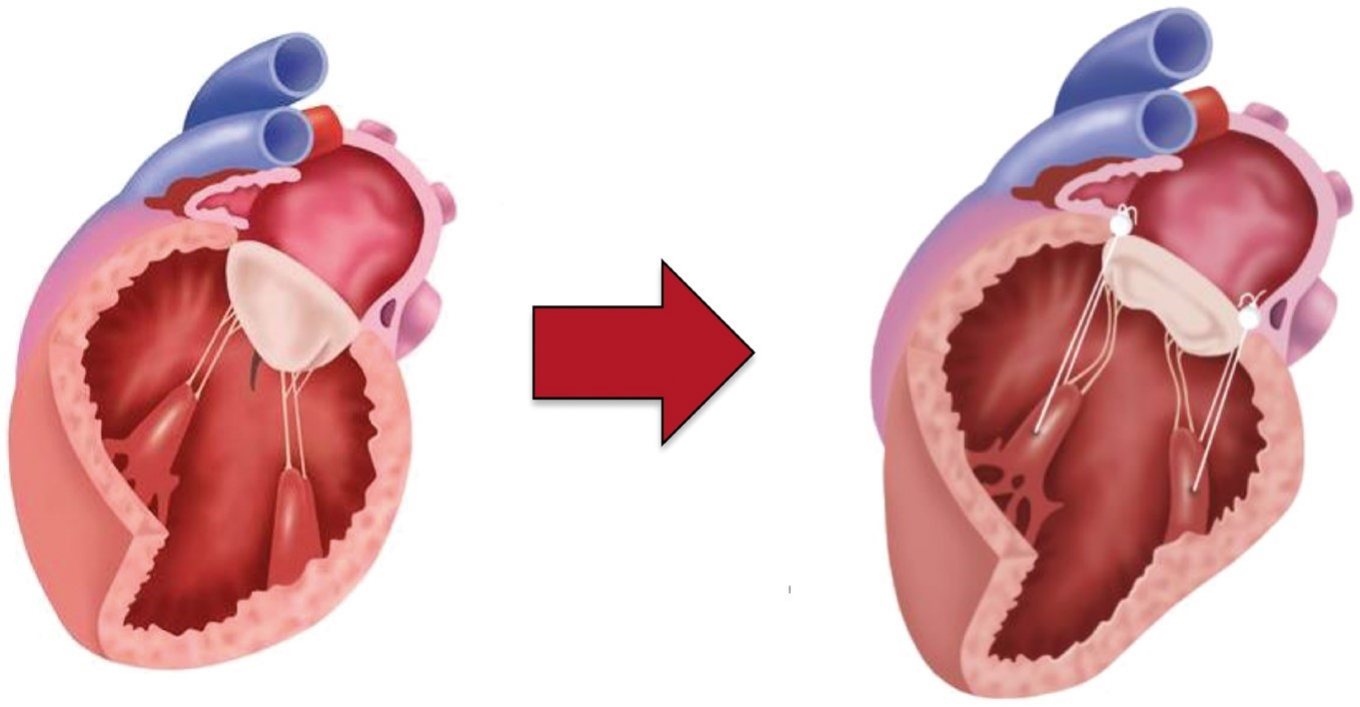
**Geometric relationship between PM tips and mitral annular plane**. increased distance with resulting leaflet tenting and reduced systolic movement towards MV annular plane (adapted from [10]). PM, papillary muscle; MV, mitral valve.

**Fig. 3.**
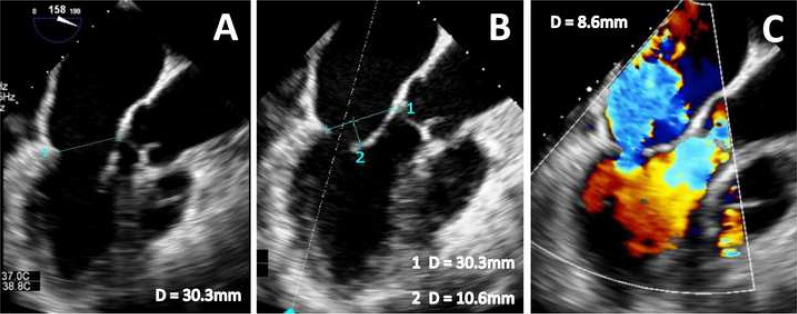
**Echocardiographic images of ventricular SMR in the setting of acute posterolateral STEMI**. TOE images show a “pure” ventricular SMR (C) caused by acute LV-remodeling due to posterolateral STEMI that results in severe tenting (tenting height 10.6 mm, B) without annular dilatation (A-P annulus 30.3 mm, A,B). STEMI, ST-elevation myocardial infarction; SMR, secondary mitral regurgitation; LV, left ventricular; TOE, transoesophageal echocardiography.

Ventricular SMR is a sequel of distorted LV geometry- global or regional, causing a displacement of papillary muscles apically and laterally (Fig. 2, Ref. [10]). Consequently, leaflet tenting occurs since the chordal length of the MV leaflets does not increase in SMR. The severity of leaflet tenting correlates linearly with the distance between papillary muscle tips and the mitral annular plane. The larger this distance gets, the more extensive leaflet tenting occurs, and the more severe the ventricular SMR is. Concomitant annular dilatation may occur in the chronic ventricular SMR and is rather a secondary finding than a primary mechanism causing SMR. In line with this, annular dilatation is almost absent in the acute ventricular SMR, e.g., in the setting of fulminant myocarditis or acute myocardial infarction (Fig. 3).

Since the pathophysiological origin of ventricular SMR is papillary muscle displacement, the treatment strategy should focus on reestablishing the appropriate papillary muscle position in relation to the MV annulus plane. Papillary muscle maneuvers actively reverse papillary muscle displacement and relieve leaflet tenting, enabling normal systolic leaflet motion and coaptation at the MV annulus level.

### 3.4 Echocardiographic Criteria of Atrial vs. Ventricular SMR 

No quantitative echocardiographic cut-off values are yet available to reliably separate atrial vs. ventricular SMR patients. Therefore, a combination of echocardiographic parameters should be considered. Echocardiographic markers of systolic LV dysfunction (i.e., depressed LVEF, reduced LV fractional shortening or global longitudinal strain) indicate rather the ventricular mechanism of SMR, as well as markedly increased LV volume and diameter measurements. However, some SMR patients present with almost normal LVEF and only slightly increased LV diameters/volumes and still have echocardiographic evidence of severe posterior mitral leaflet (PML) tethering and/or seagull sign in the anterior mitral leaflet (AML) indicating ongoing LV remodeling. The echocardiographic signs of leaflet tethering (i.e., PML angle, tenting height and tenting area) are the surrogate parameters of increased papillary muscle tips to MV annular distance and, therefore, the markers of ventricular component in the SMR development. The resultant regurgitant jet is often eccentric and imitates AML prolapse (so-called AML pseudoprolapse). When treating such patients, surgical maneuvers to address papillary muscle to MV annulus distance should be strongly considered. As opposite, an echocardiographic finding of normal systolic leaflet movement, in combination with a central regurgitant jet in the mid-part of the mitral valve or along the entire coaptation line, is supportive of the atrial origin of SMR. In particular, if the abovementioned findings coincide with a markedly dilated MV annulus and nearly preserved LV geometry. Such patients can be safely treated by an annuloplasty-only approach. However, one should keep in mind that these parameters are only valid in an awake patient under physiologic preload and afterload conditions. Therefore, the evaluation of the SMR mechanism can be misleading when transoesophageal echocardiography (TOE) is performed under general anesthesia.

### 3.5 Indications for Papillary Muscle Maneuvers in SMR

LV remodeling is routinely assessed by LV size and volume indices [11]. Left ventricular end-diastolic diameter (LVEDD), end-systolic and end-diastolic volume indices (i.e., left ventricular end-systolic volume index (LVESVI), left ventricular end-systolic volume index (LVEDVI)) as well as LV sphericity index (LVSI) are routinely used to quantify LV remodeling severity and to compare outcomes of SMR intervention [11]. LV size/volume correlates significantly with the clinical outcome [12] as well as MR reoccurrence after MV repair in SMR [13]. However, there is no evidence for a linear correlation between LV size/volume and SMR severity [14]. This underlines the role of extensive papillary muscle displacement even in “localized” LV remodeling causing severe SMR, which is not captured by “global” LV size/volume parameters. Therefore, even though LV size/volume indices describe the severity of global LV remodeling, they are insufficient to solely defining the necessity of papillary muscle maneuvers in SMR patients.

There might be two potential ways to better define the ventricular SMR mechanism and thereby the need of papillary muscle maneuvers. The severity of leaflet tenting correlates linearly with the degree of papillary muscle displacement and SMR severity [14]. Previous studies showed a strong association between the severity of leaflet tenting (e.g., preoperative tenting area) and SMR reoccurrence after MV repair [15, 16]. Nappi *et al*. [15] evaluated preoperative echocardiographic characteristics associated with recurrent MR at 5 years following MV repair and showed that a baseline tenting area ≥3.1 ${\mathrm{cm}}^{2}$ was associated with a recurrent MR. Another study by Karaca *et al*. [16] revealed that an MV tenting area >3.4 ${\mathrm{cm}}^{2}$ was associated with a worse functional status, more hospitalizations, and higher mortality when compared to a tenting area <3.4 ${\mathrm{cm}}^{2}$. Therefore, an MV tenting area could be a quantitative marker indicating the predominant ventricular mechanism of SMR and thereby the need for papillary muscle maneuvers. On the other hand, tenting might be asymmetric (e.g., most severe tenting at the posteromedial commissure) and, therefore, the two-dimensional definition of tenting area is suboptimal. Therefore, the definition of tenting volume by three-dimensional echocardiography could be more appropriate and needs to be validated [17].

Another option to define the predominant ventricular origin of SMR and the need for papillary muscle maneuvers is to directly measure the displacement of the papillary muscles from an MV annular plane by means of papillary muscle to mitral annulus distance (PMAD) [18]. When indexed to global LV size/volume, PMAD may define the “ideal” distance between papillary muscle tips and MV annulus plane for the individual LV geometry and thereby guide papillary muscle maneuvers during SMR treatment [18]. There is ongoing research to validate echocardiographically measured PMAD as a guiding tool for papillary muscle maneuvers in SMR treatment.

### 3.6 Evidence for Papillary Muscle Maneuvers in SMR

The idea of alternative approaches in ventricular SMR emerged from the dismal results of isolated annuloplasty [19, 20]. Due to frequent failure of isolated annuloplasty, there was a shift towards MV replacement in ventricular SMR, abolishing the problem of recurrent MR. However, MV replacement in ventricular SMR was consistently associated with 2.0–2.5 times higher perioperative mortality as compared to MV repair [21, 22, 23]. Although a CTS-Net trial by Acker *et al*. [24] showed no significant difference in 2-year survival between MV replacement and MV repair in ischemic SMR patients [20], reverse LV remodeling as determined by LVESVI was more prominent with a durable MV repair (i.e., LVESVI 42.7 ± 26.4 mL/$m^{2}$ (durable MV repair) vs 60.6 ± 39.0 mL/$m^{2}$ (MV replacement), *p* < 0.0001). This finding indicates the necessity of durable MV repair in ventricular SMR.

Papillary muscle maneuvers that reestablish normal papillary muscle geometry, relieve leaflet tenting and restore normal coaptation, may improve the long-term stability of MV repair [25]. A previous meta-analysis compared the reoccurrence of MR >2 after MV repair in ventricular SMR patients undergoing isolated annuloplasty vs. annuloplasty with additional ventricular repair maneuvers [26]. The combination of annuloplasty with ventricular repair was associated with a fourfold lower reoccurrence of MR >2 as compared with the annuloplasty alone (odds ratio, OR 0.27, 95% CI 0.19–0.38, *p* < 0.0001) [26]. A randomized trial by Nappi *et al*. [27] evaluated long-term outcomes of ventricular SMR patients who underwent isolated annuloplasty vs. annuloplasty with papillary muscle maneuvers. At 5 years, papillary muscle maneuvers demonstrated better LV re-remodeling and lower MR >2 reoccurrence as compared to annuloplasty alone [27]. Another prospective study that compared standardized papillary muscle maneuvers vs. annuloplasty alone in ventricular SMR patients, revealed significantly reduced MR >2 reoccurrence and improved clinical outcomes at 1-year [28]. This single center data was confirmed by recent findings of the multicenter REFORM-MR registry that showed excellent outcomes of standardized papillary muscle maneuvers in ventricular SMR patients [29].

### 3.7 Techniques of Papillary Muscle Maneuvers

Historically, a wide variety of adjuncts to annuloplasty were used in ventricular SMR. Leaflet augmentation has been used to increase the surface of the posterior or anterior mitral leaflet and thereby mitigating the effect of leaflet tenting [30]. Important drawbacks of this technique are: (I) no impact LV geometry; (II) patch suturing is a time-consuming procedure in the setting of severe LV dysfunction; (III) the patch material is prone to bio-degeneration, leading to restriction/calcification [31]; (IV) patch may tear from the native leaflet tissue thereby inducing an acute MR [32]. Secondary chordae cutting has been previously used to reduce anterior mitral leaflet tenting [33]. However, only tethering in the belly of the anterior mitral leaflet (i.e., seagull sign) can be addressed by this technique, while the free edge of the anterior mitral leaflet remains tethered. Therefore, this technique is effective only in patients with mild-to-moderate tethering [34]. Furthermore, the impact of secondary chordae cutting on LV remodeling remains unclear.

**Fig. 4.**
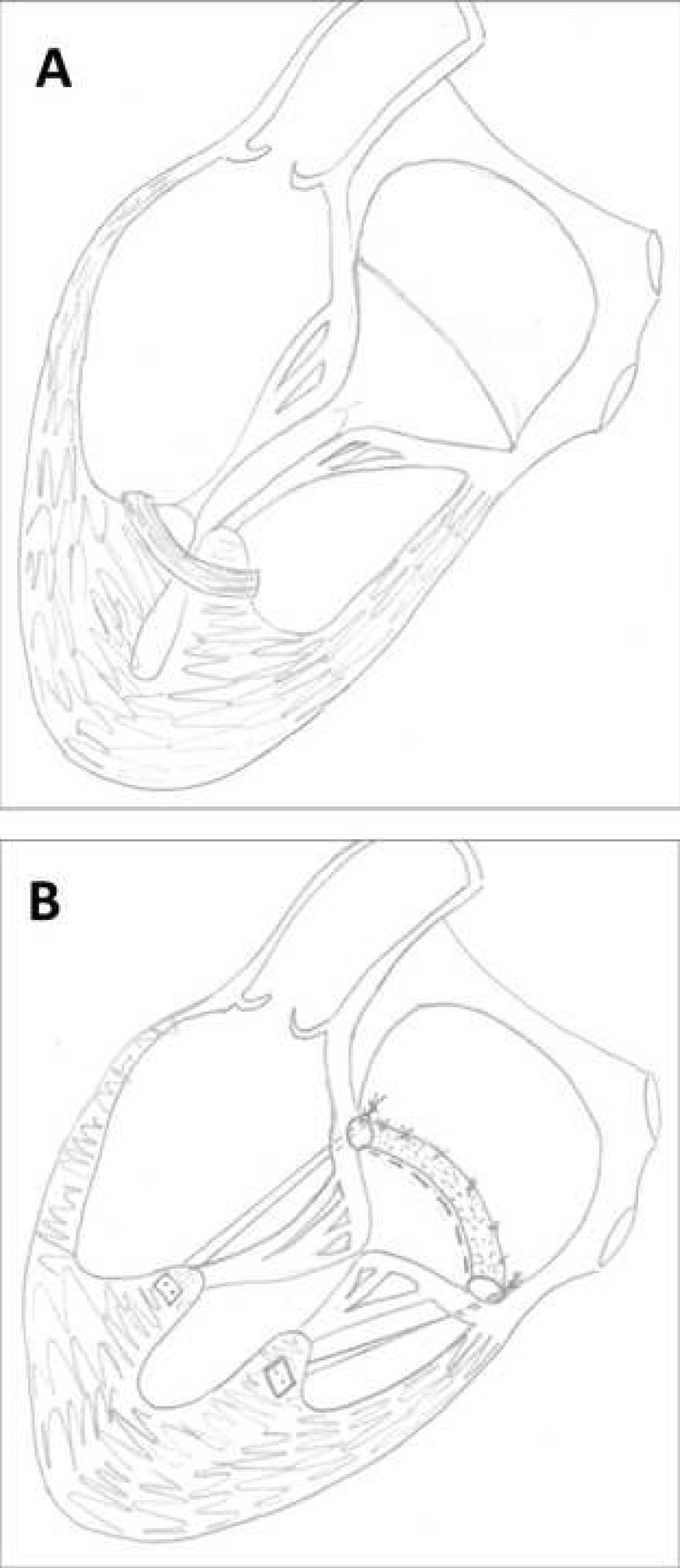
**Papillary muscle maneuvers**. (A) Papillary muscle reapproximation to correct lateral papillary muscle displacement; (B) Papillary muscle repositioning to predominantly address apical papillary muscle displacement.

Given these limitations, the focus constantly moved toward papillary muscle maneuvers. Papillary muscle maneuvers represent the most frequently used technique to address MV leaflet tenting [35]. The key idea behind these techniques is to re-establish the normal geometric position of both papillary muscles with regard to the MV annular plane and thereby enable normal systolic leaflet coaptation at the annulus level. By counteracting papillary muscle displacement in a remodeled LV, papillary muscle maneuvers represent the pathophysiological approach to treat ventricular SMR. Papillary muscle maneuvers may be subdivided into (a) papillary muscle re-approximation that brings both papillary muscles together and, therefore, addresses the lateral papillary muscle displacement (Fig. 4A) (e.g., suture-based papillary muscle approximation [35], papillary muscle sling [27]), and (b) papillary muscle repositioning that reduces the distance between papillary muscle tips and mitral annular plane and, therefore, predominantly corrects the apical papillary muscle displacement (Fig. 4B) [10, 36]. Papillary muscle maneuvers provide the highest freedom from recurrent MR >2 (i.e., 95% at 3-year follow-up) as compared to other SMR treatment techniques [26]. However, no prospective studies are comparing different papillary muscle maneuvers, and, therefore, scientific evidence for the most appropriate technique is still lacking.

Several practical aspects are important when choosing papillary muscle maneuver to treat ventricular SMR. First of all, the technique should be simple and reproducible to enable its quick adoption by most MV surgeons. Second, papillary muscle maneuver should be expeditious to not substantially increase myocardial ischemia time in patients with severe LV dysfunction. Third, it should be applicable in every clinical scenario, including minimally invasive MV surgery. Finally, the papillary muscle maneuver should have the potential to develop further into a catheter-based technique.

### 3.8 Papillary Muscle Maneuvers in Ischemic vs. Non-ischemic Cardiomyopathy

Lesions leading to LV remodeling and occurrence of ventricular SMR are heterogeneous and can be grossly separated into ischemic and non-ischemic injuries. Although an inferior myocardial infarction may initially result in an isolated posteromedial papillary muscle (PM) dysfunction and/or regional LV remodeling with resultant asymmetric MV leaflet tethering, the subsequent pathophysiological pathway of LV disease progression is quite similar to a primarily global LV injury, i.e., in the setting of dilated cardiomyopathy. The ongoing LV remodeling is followed by progressive papillary muscle displacement and increasing leaflet tethering, irrespective of the primary LV injuring mechanism. Therefore, papillary muscle maneuvers are performed identically in both ischemic and non-ischemic cardiomyopathies and have been shown to result in very comparable 1-year outcomes [37]. 1-year clinical and echocardiographic outcomes were similar in ischemic vs. non-ischemic ventricular SMR patients, with the only exception that LVEDD was significantly reduced in the non-ischemic subgroup but not in the ischemic subgroup. Of note, freedom of MR ≥2 at 1 year postoperatively was low and comparable in both groups [37].

## 4. Advancing the Treatment of Ventricular SMR

Despite continuous refinements in MV therapies, SMR remains a permanent challenge and the best treatment strategy has still to be defined, as demonstrated recently by 5-year COAPT results [38]. Multiple efforts to identify the responders to MV intervention in SMR led to the definition of some most appropriate clinical and echocardiographic phenotypes [3]. However, room for major improvement exists and an individualized SMR treatment protocol should consider the following patient-specific issues:

### 4.1 Is there a Relevant MR to Benefit from Intervention?

The definition of severe SMR is still a matter of controversy, as illustrated by changing cut-off values in the European (ESC/EACTS) [39] and American (AHA/ACC) [40] guidelines for management of Valvular Heart Disease. Patients with a moderate SMR do not seem to benefit from MV intervention [41], however, how is moderate SMR defined? The quantification of SMR is aggravated by the fact that SMR is a highly dynamic condition and its severity changes significantly depending on LV geometry. An increase in cardiac output and changes in the heart volume status may provoke further LV distension, an increase in tethering severity and consequently aggravation of SMR [42]. The systematic use of exercise stress echocardiography to unmask severe SMR is insufficiently standardized and, therefore, underused and understated in the guideline recommendations. Furthermore, the value of three-dimensional (3D) echocardiography and CMR to identify patients with severe SMR when two-dimensional (2D) echocardiography is inconclusive remains to be defined [43].

### 4.2 Quantification of LV Disease Severity

This is a key question to select the appropriate candidates for SMR intervention. Some data demonstrates that SMR patients with so-called moderate/intermediate heart failure phenotypes benefit the most from the MV intervention [3]. However, the use of standard size- and volume-based echocardiographic parameters to predict the severity of LV disease is limited due to highly dynamic LV preload and afterload conditions. Furthermore, the use of LVEF as a quantitative marker of LV disease severity may be misleading in the setting of severe SMR. There is increasing evidence for the importance of LV global longitudinal strain to quantify the severity of LV disease [44]. Ideally, the measurement of global intramyocardial fibrosis burden will be used in the future to predict the chance of prognostic benefit of MV treatment in ventricular SMR patients [45].

### 4.3 Durability of MV Intervention

Based on the results of a previous randomized trial [20] and ACC/AHA guideline recommendations [40] a chordal-sparing MV replacement theoretically provides the most durable relief of ventricular SMR. Despite the theoretical advantages of durable MR relief, MV replacement in ventricular SMR is a high-risk procedure, associated with prohibitive perioperative mortality/morbidity in the surgical and catheter-based cohorts [21, 22, 23, 46]. Therefore, durable MV repair is a highly relevant issue in ventricular SMR. Isolated annuloplasty results in an unacceptable rate of recurrent MR in ventricular SMR patients [20]. TEER is similarly associated with a significant MR >2 reoccurrence rate, as demonstrated in >30% of patients at 6-month echocardiographic follow-up in the COAPT trial [38]. Therefore, papillary muscle maneuvers that target the pathophysiological mechanism of ventricular SMR seem to be one of the most promising therapeutic strategies in the future to enable a durable MV repair [26].

## 5. Conclusions

SMR treatment strategies are evolving, however the most appropriate SMR intervention remains to be defined. Papillary muscle maneuvers target the pathophysiological mechanism of ventricular SMR and provide an upcoming therapeutic tool in ventricular SMR patients. Such surgical techniques in well-selected patient subgroups with a ventricular SMR have clear potential to improve long-term outcomes in SMR treatment. Multicenter randomized trials comparing papillary muscle maneuvers with the chordal-sparing MV replacement and catheter-based techniques in well-defined ventricular SMR populations is the next logical and inevitable step.
